# Whole-Body Vibrations Do Not Elevate the Angiogenic Stimulus when Applied during Resistance Exercise

**DOI:** 10.1371/journal.pone.0080143

**Published:** 2013-11-15

**Authors:** Åsa Beijer, André Rosenberger, Birgit Bölck, Frank Suhr, Jörn Rittweger, Wilhelm Bloch

**Affiliations:** 1 German Aerospace Center, Institute of Aerospace Medicine and Space Physiology, Cologne, Germany; 2 German Sport University Cologne, Department of Molecular and Cellular Sport Medicine, Cologne, Germany; 3 German Sport University Cologne, Institute of Training Science and Sports Informatics, Cologne, Germany; 4 Institute for Biomedical Research into Human Movement and Health, Manchester Metropolitan University, Manchester, United Kingdom; Ottawa Hospital Research Institute, Canada

## Abstract

Knowledge about biological factors involved in exercise-induced angiogenesis is to date still scanty. The present study aimed to investigate the angiogenic stimulus of resistance exercise with and without superimposed whole-body vibrations. Responses to the exercise regimen before and after a 6-week training intervention were investigated in twenty-six healthy male subjects. Serum was collected at the initial and final exercise sessions and circulating levels of matrix metalloproteinases (MMP) -2 and -9, Vascular Endothelial Growth Factor (VEGF) and endostatin were determined via ELISA. Furthermore, we studied the proliferative effect of serum-treated human umbilical vein endothelial cells *in vitro* via BrdU-incorporation assay. It was found that circulating MMP-2, MMP-9, VEGF and endostatin levels were significantly elevated (*P*<0.001) from resting levels after both exercise interventions, with higher post-exercise VEGF concentrations in the resistance exercise (RE) group compared to the resistive vibration exercise (RVE) group. Moreover, RE provoked increased endothelial cell proliferation *in vitro* and higher post-exercise circulating endostatin concentrations after 6 weeks of training. These effects were elusive in the RVE group. The present findings suggest that resistance exercise leads to a transient rise in circulating angiogenic factors and superimposing vibrations to this exercise type might not further trigger a potential signaling of angiogenic stimulation in skeletal muscle.

## Introduction

There is growing evidence that physical activity is a potent stimulator of angiogenesis in skeletal and cardiac muscle [1]. Endurance training is thought to increase capillarity in skeletal muscle [2], whereas high resistance training has been shown to decrease capillary density [3], most likely as a result of fibre hypertrophy with insufficient angiogenesis. Knowledge about the exact mechanisms of blood vessel growth is to date still scanty. In the current models of sprouting angiogenesis, capillary formation involves two essential steps, namely (i) degradation of the extracellular matrix (ECM) surrounding the capillary and (ii) activation, migration and proliferation of capillary endothelial cells [4].

ECM breakdown is mediated by a family of zinc- and calcium-dependent enzymes, the matrix metalloproteinases (MMP) [5]. The proteases MT1-MMP, MMP-2 and -9 seem to play a crucial role in the formation of new capillaries in skeletal muscle [6] and previous studies reveal that their serum concentrations are significantly elevated after endurance exercise [7]. Furthermore, members of the MMP-family are known to release endostatin by proteolytic cleavage of the C-terminal NC1 domain of Collagen XVIII [8]. To date, the role of endostatin in the angiogenic process is not clear due to its complex signaling functions. As both pro-angiogenic [9] and anti-angiogenic [10] characteristics have been described for endostatin, it has been considered to function as an angiogenic modulator [11]. Endostatin seems to play a crucial role in exercise-induced angiogenesis, as serum concentrations were acutely elevated after endurance exercise [12], [13]. However, other studies have reported decreased serum concentrations of endostatin as an adaptation to long-term endurance training [7].

Endothelial cell activation, migration, and proliferation is mediated by Vascular Endothelial Growth Factor (VEGF), a potent endothelial cell mitogen [14]. VEGF has been shown to be activated upon elevated shear stress perturbation [15], muscle stretch [16] and hypoxia [17]. Additionally, VEGF has been reported to be essential for exercise-induced angiogenesis in skeletal muscle [18]. The findings of a previous study evaluating the effects of endurance exercise with and without whole-body vibrations revealed that circulating VEGF was specifically increased in the group where vibrations were superimposed to the exercise stimulus [13].Of note, it has been suggested that the mechanical stimulus of whole-body vibration (WBV) increases shear stress at the walls of blood vessels [19], leads to increases in blood flow velocity after vibration termination [20] and can elicit muscle de-oxygenation [21]. Based on the finding that shear stress and hypoxia are able to induce angiogenesis [4], we hypothesized that the superposition of a vibration stimulus to resistance exercise would add a pro-angiogenic stimulus to the exercise. It would be desirable to find a novel training mode that concurrently increases muscle strength and induces capillary growth to optimize the flux of oxygen and nutrients to the muscle and thus improve muscular performance. In order to investigate the pro-angiogenic stimulus of the exercises, we determined serum concentrations of the angiogenic factors MMP-2, MMP-9, VEGF and endostatin at rest and in response to resistance exercise and resistive vibration exercise. Additionally, we performed *in vitro* assays to evaluate the proliferative property of exercise-serum treated endothelial cells.

## Materials and Methods

### Ethics statement

Twenty-six healthy, recreationally active male subjects (26±0.8 years) were included into the study after providing a written informed consent. The study was conducted in compliance with the *Declaration of Helsinki* following approval by the Ethics Committee of the Northern Rhine medical association (Ärztekammer Nordrhein) in Düsseldorf (application no. 2010-174).

### Study design and subject characteristics

The present EVE study (“molecular and functional **E**ffects of **V**ibration **E**xercise”) was conducted in a stratified, randomized two-group parallel design. A detailed description of the exercises and study design has been published elsewhere [22]. Any competitive sports, participation in strength training during the past six months, smoking, diabetes as well as any current medication were considered as exclusion criteria. Subjects were stratified into two matched groups according to their maximum jumping height, forming two groups with comparable neuromuscular fitness [23]. A coin was then tossed to randomly assign the groups to one of the two training interventions: resistance exercise or resistive vibration exercise. The subjects anthropometric data at baseline are given in Table 1, and no statistically significant group difference was found (*P*>0.11).

**Table 1:** Anthropometric data of EVE subjects at baseline.

	RE group (n = 13)	RVE group (n = 13)	*P*- value
Age [yrs]	23.4 (±0.39)	24.3 (±0.92)	0.52
Body mass [kg]	72.2 (±1.30)	74.7 (±1.91)	0.89
Height [m]	1.79 (±0.01)	1.79 (±0.01)	0.31
BMI	23.4 (±0.39)	23.5 (±0.58)	0.11
CMJ height [cm]	42.2 (±1.28)	41.7 (±0.61)	0.97
Maximal performance on cycle ergometer test [W/kg body weight]	3.3 (±0.08)	3.3 (±0.11)	1.00

BMI: Body Mass Index, CMJ: Counter movement jump. There was no difference between the two groups. Values are means ± SEM

### Training design

The present study was designed to compare acute and long-term effects of two training interventions: resistance exercise (RE) and resistive vibration exercise (RVE). Participants trained 2–3 times per week for six weeks (completing 16 exercise sessions), with each session lasting 9min. Participants trained with weights on a guided barbell (PTS Dual action Smith, Hoist, U.S.A). The individual training load was set at 80% of their One-Repetition-Maximum (1-RM), which was determined according to the method described by Baechle and Earle [24].

The exercise consisted of squats (with each 2 sec. eccentric and 2 sec. concentric phase) and heel raises (with each 1 sec. eccentric and 1 sec. concentric phase), divided by a 1-min break. Movement rhythm was guided by a metronome. Each exercise session consisted of a warm-up composed of two sets with each 10 squats and 15 heel raises with the unloaded barbell (15 kg) as training weight. The actual exercise was carried out in three sets: first and second sets were composed of 8 squats ( = 32 sec. per set) and 12 calf raises ( = 24 sec. per set) and in the third set, maximum number of repetitions for squats and calf raises were performed. The subjects in the RVE group performed the resistance exercise protocol with simultaneous side-alternating whole-body vibrations (Galileo® Fitness, Novotech, Germany) with a 6 mm peak-to peak displacement, whereas subjects in the RE group trained with the same setting, without superimposed vibrations.

The training followed an incremental training design with regards to weight and vibration frequency. Training weights were increased over time according to the subjects' individual training progressions, as described previously [22]. In brief, the number of squats in the 3^rd^ set was used as a reference to re-determine the subjects' individual 80% of the 1-RM for the following training, using the method described by Baechle and Earle [24]. Training weights in the RE group increased from 75.2±1.8 kg during the initial exercise to 130.2±5.1 kg during the final exercise. Weight increase was significantly smaller the RVE group, which increased from 81.5±2.1 kg during the initial exercise to 110.2±4.4 kg during the final exercise. Training weight increase was hampered by training with vibration frequencies above 35 Hz, as discussed in the methodological paper on the training design previously published [22].

Vibration frequencies were increased from 20 Hz in the first week to 40 Hz during the last two weeks with 5-Hz weekly increments. The reason for the increase in vibration frequency was that we aimed to test physiological responses when exercising at 40 Hz side-alternating WBV, which to the best of our knowledge has not been tested in any other study. Pilot testing revealed that resistance exercise with 40 Hz side-alternating WBV is more challenging for people not accustomed to WBV, suggesting that it could potentially elicit greater effects than lower vibration frequencies, but also that one must envision problems when embarking directly on such high a frequency. Thus, in order to prevent problem-related drop-out from the RVE group and thus a study bias, we decided to initially set the vibration frequency to 20 Hz and to gradually increase the vibration frequency to 40 Hz.

### Serum Collection

**Figure 1:**
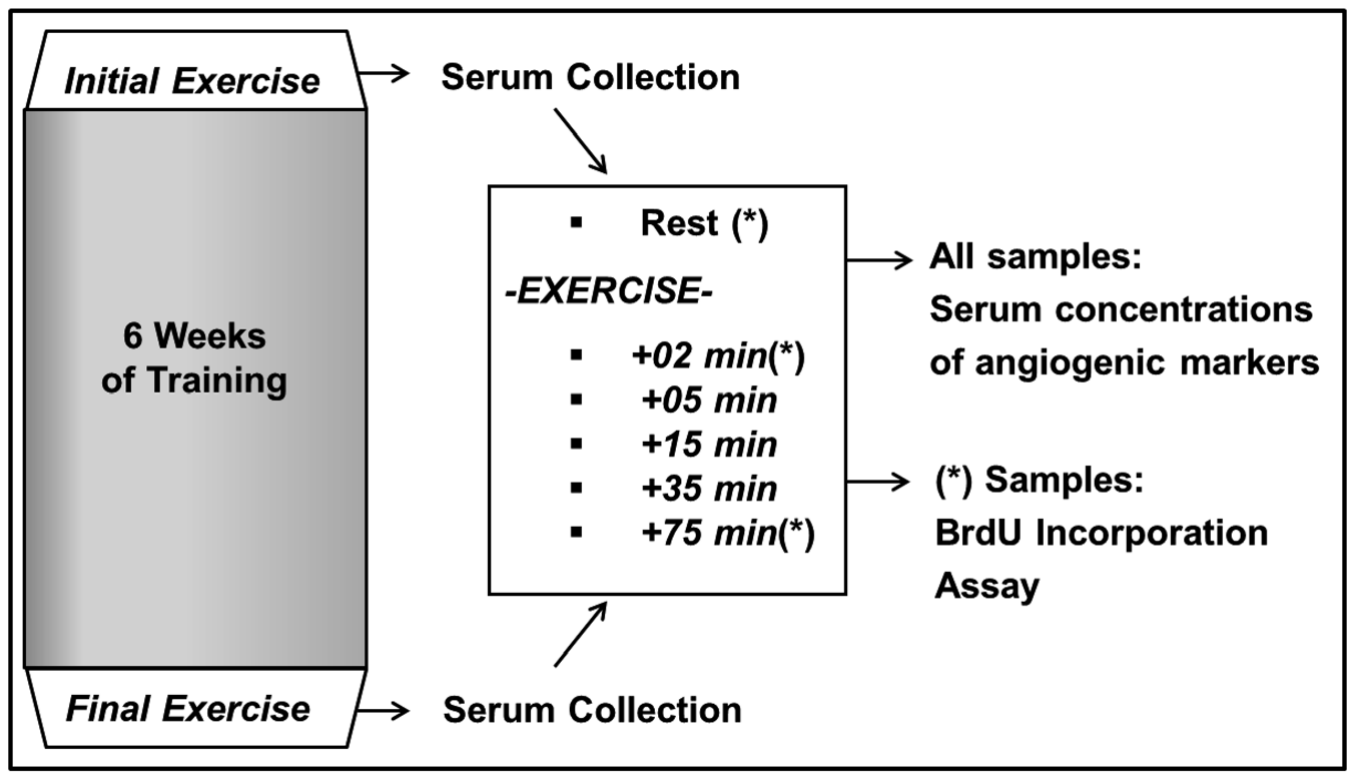
Study Design. Serum was collected at the initial and final exercise sessions of a 6-week training intervention. Time points of serum collection were 1 hour prior to exercise (Rest) and 2, 5, 15, 35 and 75 minutes after exercise termination. Serum concentrations of angiogenic markers (MMP-2, MMP-9, VEGF and endostatin) were determined for all serum samples, BrdU incorporation assay was performed with the serum samples indicated with (*): Rest, +*2 min* and +*75 min*.

Venous blood samples were collected at the initial and final exercise sessions of the 6-week training intervention as illustrated in Figure 1. On that day, subjects had a standardised breakfast (two wheat bread rolls with butter and jam) two hours before exercise. Blood was collected one hour prior to exercise (Rest) and +2 min, +5 min, +15 min, +35 min and +75 min after exercise through a short catheter into serum monovettes (Sarstedt, Nümbrecht, Germany) from the cephalic vein, allowed to clot for 10 minutes, centrifuged at 3000 rpm at 4°C (Heraeus Multifuge 1S-R, Thermo Scientific, Waltham, MA, USA), distributed into small tubes and immediately frozen at −20°C until analysis.

### ELISA analyses

Serum levels of MMP-2 (free pro- and active MMP-2 [ng/mL]), MMP-9 (92 kDa pro-MMP-9 and 82 kDa active MMP-9 isoforms [ng/mL]), VEGF (total VEGF [pg/mL]) and endostatin (total endostatin [ng/mL]) were detected in double determinations using Enzyme-linked Immunosorbent Assay (ELISA) kits (R&D Systems, Wiesbaden, Germany) according to the manufacturer's instructions.

### Cell lines and culture conditions

Human Umbilical Vein Endothelial Cells (HUVEC, #C-12200, PromoCell, Heidelberg, Germany) were cultured at 37°C and 5% CO_2_ in basal medium with added growth supplements (Endothelial Cell Growth Medium KIT, #C-22110, PromoCell, Heidelberg, Germany). Prior to incubation with human serum and 5-Bromo-2-Deoxyuridine (BrdU), cells were split into 96-well plates (DetachKit, #C-41210, PromoCell, Heidelberg, Germany) and cultured in starvation medium (i.e. basal medium with only 0.5% Fetal Calf Serum as growth supplement) for 24 hours. BrdU incubation was performed in conditioned medium (i.e. basal medium containing 2% of human serum providing growth and proliferation factors). Sera obtained from pre- and post- exercise (Rest, +*2 min* and +*75 min* post) at each initial and final exercise sessions were used for generating the conditioned medium, see Figure 1.

### BrdU incorporation assay

Samples were incubated with BrdU for 20 hours and detection of BrdU incorporation was performed in double determinations via ELISA (BrdU Cell Proliferation Assay Kit, #6813, Cell Signaling Technology, Danvers, MA, USA) according to the manufacturer's instructions.

### Statistical Analyses

Statistical analyses were performed using STATISTICA 10 for Windows (Statsoft, Tulsa, Oklahoma, USA, 1984-2010). The effect of either resistance exercise (RE) or resistive vibration exercise (RVE) on serum concentrations of the angiogenic factors MMP-2, MMP-9, VEGF and endostatin was determined via repeated measures ANOVA with time (Rest *vs.*+*2 min*,+*5 min*,+*15 min*,+*35 min*, +*75 min* after exercise) and training status (initial *vs.* final exercise session) as factors. BrdU incorporation data were normalised to fold increases from resting levels (i.e. absorption of cells incubated with serum derived +*2 min* and +*75 min* after exercise divided by absorption of cells incubated with serum at *Rest*). A repeated ANOVA was performed with time (*+2 min vs.+75 min*) and training status (initial *vs.* final exercise) as factors. Tukey's test was used for post-hoc testing. Values are given as means ± standard error of means (SEM). Statistical significance level was set at *P*<0.05.

## Results

### Resting levels

Resting levels of the circulating angiogenic factors MMP-9, VEGF and endostatin were comparable before and after the 6-week training intervention (*P*>0.19) and there were no significant differences in resting levels between the two groups (*P*>0.68), as shown in Table 2. Resting levels of MMP-2 measured at the final exercise session differed between groups with the RVE group depicting higher values than the RE group (RVE: 193.0±8.71 ng/mL *vs*. RE: 172.0±8.5 ng/mL, *P*<0.001), which had not been the case at the initial exercise session (*P* = 0.37).

**Table 2:** Resting levels of angiogenic markers measured at the initial and final exercise sessions of the 6-week training intervention.

	RE		RVE	
	Initial exercise	Final exercise	Initial exercise	Final exercise
MMP-2 [ng/mL]	181±9	172±8	186±6	193±8^###^
MMP-9 [ng/mL]	231±17	218±19	203±21	224±35
VEGF [pg/mL]	234±53	242±50	211±37	216±38
Endostatin [ng/mL]	102±4	99±5	105±3	103±4

There were no differences in resting levels between the RE and RVE group for MMP-9, VEGF and Endostatin (*P*>0.68). After the 6-week training intervention, the RVE group had significantly higher MMP-2 levels compared to the RE group (^###^ *P*<0.001). RE: resistance exercise, RVE resistive vibration exercise MMP: Matrix metalloproteinase, VEGF: Vascular Endothelial Growth Factor. Values are means ± SEM.

### Effect of Resistance Exercise upon angiogenic factors

MMP-2, MMP-9, VEGF and endostatin were all significantly increased from resting levels after both resistance exercise and resistive vibration exercise (time effect: *P*<0.001) and all factors depicted maximum concentrations two minutes after exercise termination. In the following, relative increases from resting levels are given for the maximum concentrations that were measured at the time point +*2 min*.

### MMP-2

#### Acute effects

**Figure 2:**
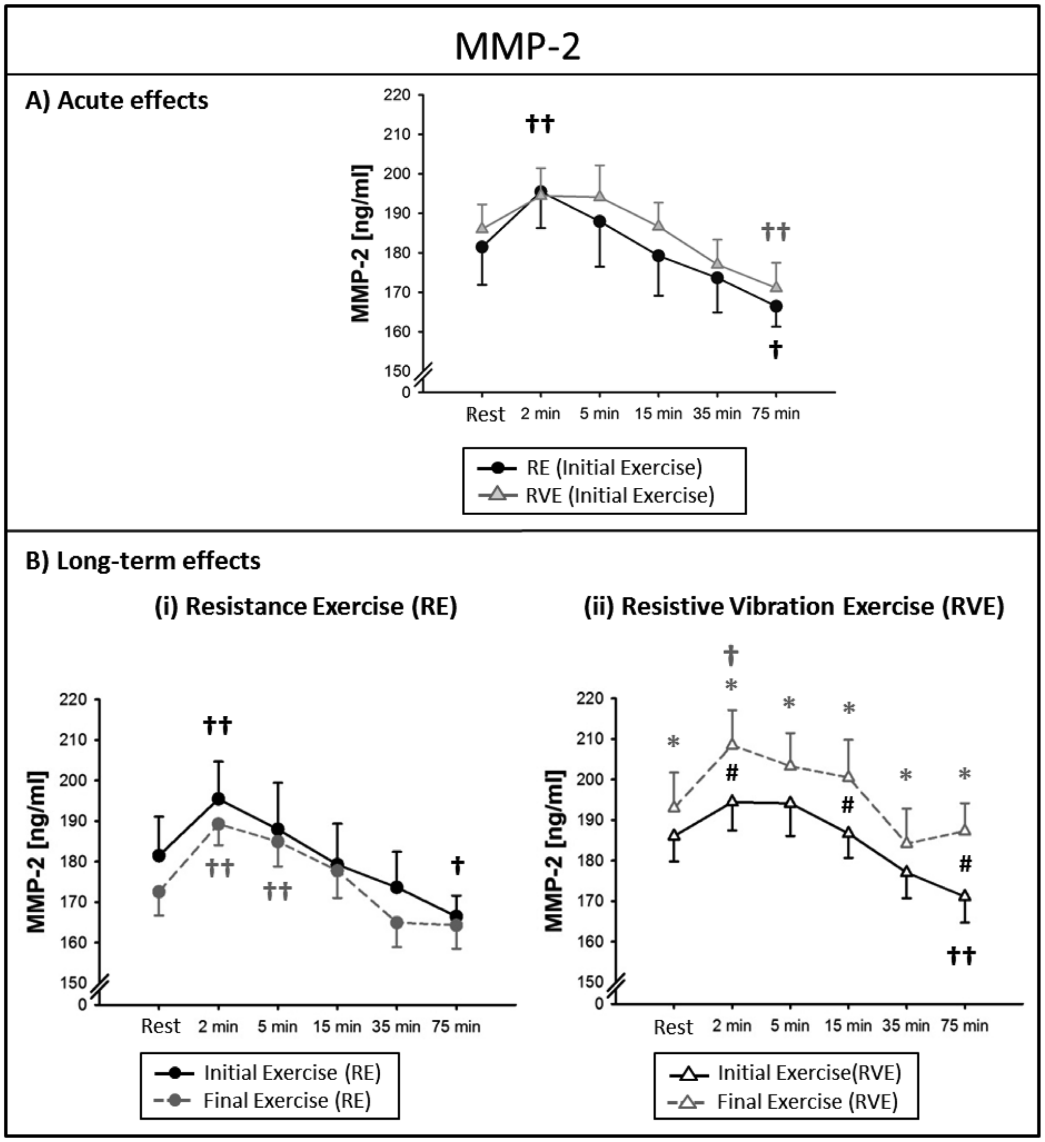
Circulating matrix metalloproteinase (MMP)-2 levels at rest and 2-75 min after exercise. Data points indicate mean serum concentrations (± SEM) at the initial and final exercise sessions of a 6-week training intervention (n = 13). Significant differences from resting levels (time effect): †*P*<0.05, ††*P*<0.001; significant differences from the initial exercise at the same time point ^#^ *P*<0.05; significant differences between groups at the final exercise *P<0.01. (A) Acute effects of resistance exercise (RE) and resistive vibration exercise (RVE): MMP-2 was elevated from resting levels only in the RE group. (B) Long-term effects: the acute response after the final exercise in the RVE group was elevated over the time course measured at the initial exercise and the RVE group depicted significantly higher MMP-2 levels at all time points compared to the RE group.

In the RE group, MMP-2 levels were increased from resting levels by 8±2% *P* = 0.001) two minutes after the initial exercise and decreased by 5±1% (*P* = 0.035) at the time point +*75 min*. In the RVE group, on the contrary, MMP-2 levels were not significantly elevated from resting levels after the initial exercise (*P* = 0.9), and were decreased by 8±2% (*P* = 0.01) at the time point+*75 min* (Fig. 2A). There were no significant differences between RE and RVE groups at the initial exercise (*P* = 0.99).

#### Long-term effects

In the RE group, there were no significant differences in the time courses when comparing initial and final exercise sessions (*P* = 0.99) as depicted in Fig. 2B(i). At the final exercise of the RVE group, however, the MMP-2 levels were generally elevated over the time course of the initial exercise (time*intervention effect: *P* = 0.049), see Figure 2B(ii). Post-Hoc testing revealed that MMP-2 concentrations were significantly higher at the time points +*2 min* (*P* = 0.028), +*15 min* (*P* = 0.019) and +*75 min* (*P* = 0.015) in the RVE group compared to the same time point at the initial exercise. While MMP-2 was not elevated from resting levels in the RVE group after the initial exercise of the 6-week training intervention, MMP-2 concentrations were significantly elevated by 8±2% (*P* = 0.02) two minutes after the final exercise. Due to the RVE-specific increases in MMP-2 concentrations, clear group differences were apparent at the final exercise session with the RVE group depicting significantly higher MMP-2 concentrations compared to the RE group at rest and after exercise (RE *vs.* RVE: *P*<0.01).

### MMP-9

#### Acute effects

**Figure 3:**
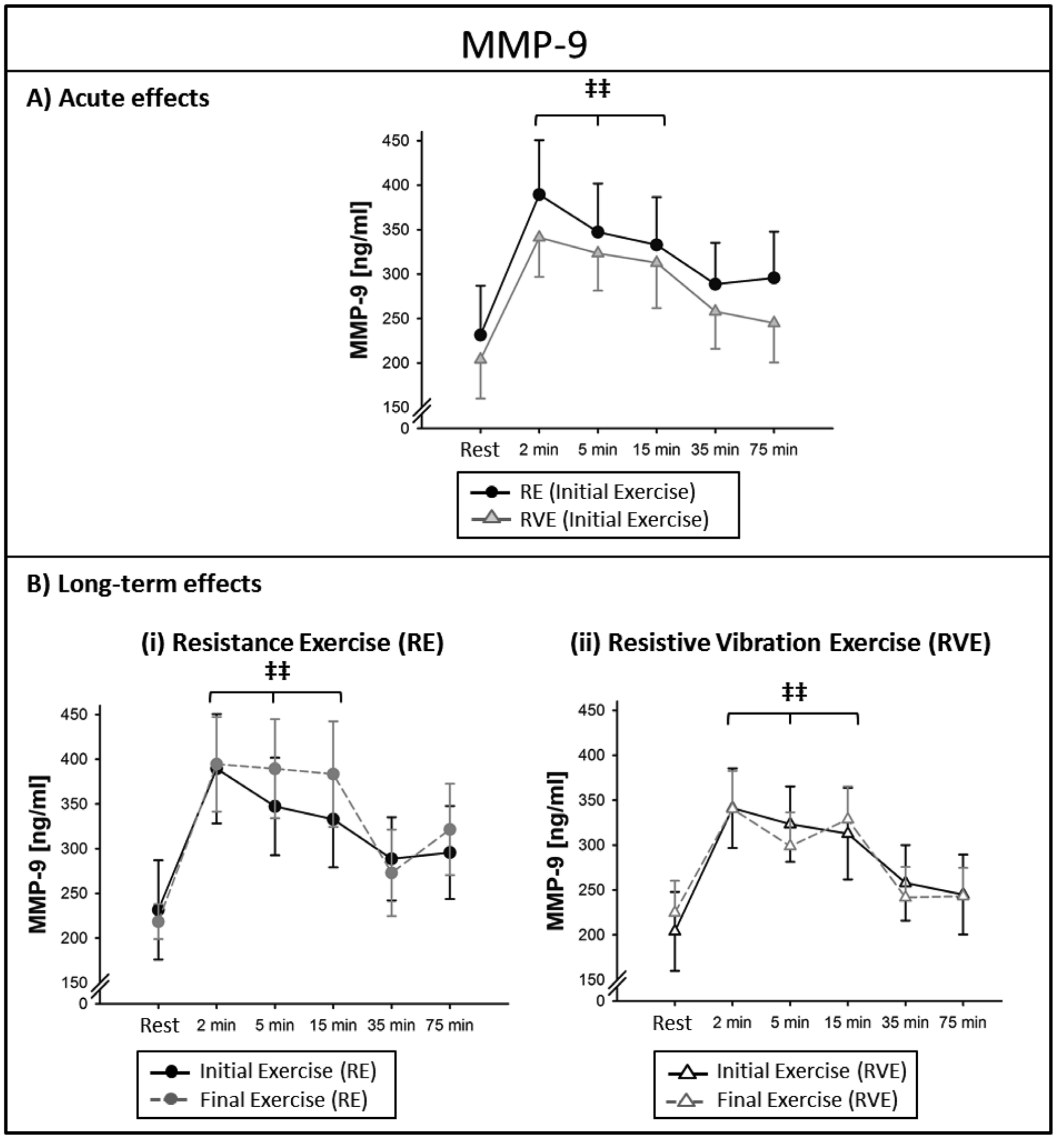
Circulating matrix metalloproteinase (MMP)-9 levels at rest and 2–75 min after exercise. Data points indicate mean serum concentrations (± SEM) at the initial and final exercise sessions of a 6-week training intervention (n = 13). (A) Acute effects of resistance exercise (RE) and resistive vibration exercise (RVE); (B) Long-term effects: In both groups, MMP-9 levels were increased over resting levels 2–15 min after exercise. Significant differences from resting levels (time effect): ^‡‡^ *P*<0.01. There were no differences between initial and final exercises of the 6-week intervention in neither group.

MMP-9 was elevated from resting levels 2–15 min after exercise (time effect: *P*<0.001). The MMP-9 increase after the initial exercise accounted for 71±19% in the RE group and 74±16% in the RVE group with no significant differences between groups (RE *vs.* RVE: initial exercise: *P* = 0.439; final exercise: *P* = 0.35), see Fig. 3A.

#### Long-term effects

There was no effect of the 6-week training intervention upon the acute MMP-9 response in serum (initial *vs.* final exercise: RE: *P* = 0.44; RVE: *P* = 0.98), see Figure 3B.

### Endostatin

#### Acute effects

**Figure 4:**
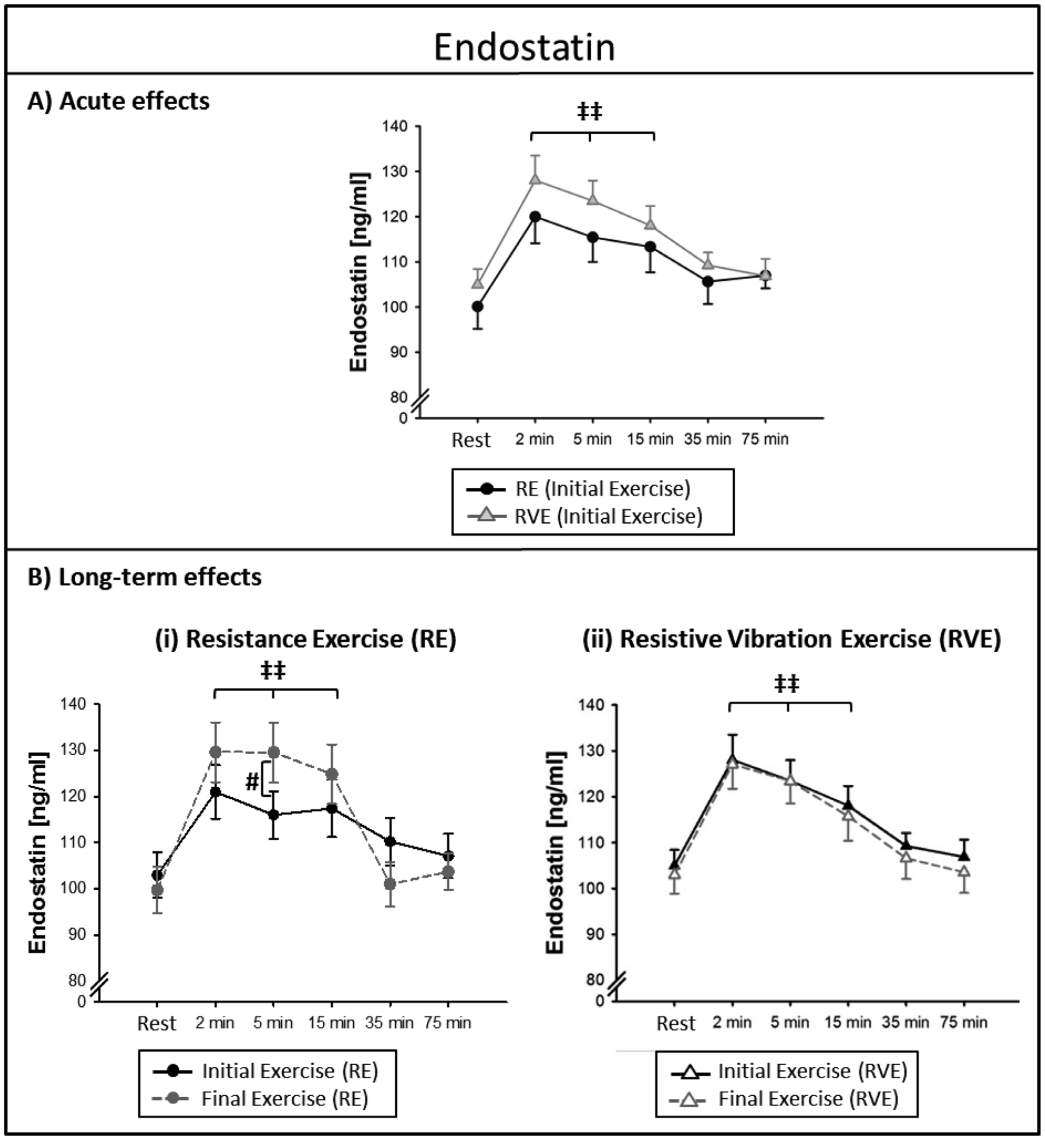
Circulating endostatin levels at rest and 2–75 min after exercise. Data points indicate mean serum concentrations (± SEM) at the initial and final exercise sessions of a 6-week training intervention (n = 13). Endostatin levels were increased over resting levels 2-15 min after training (time effect): ^‡‡^ *P*<0.01. (A) Acute effects of resistance exercise (RE) and resistive vibration exercise (RVE): the acute exercise effects did not differ between groups. (B) Long-term effects: circulating post-exercise endostatin levels in the RE group were higher at the final exercise compared to the initial exercise: ^#^ *P*<0.05.

Serum levels of endostatin were increased from resting levels 2–15 min after both RE and RVE (time effect: *P*<0.001). After the initial training, endostatin levels were elevated by 17±3% in the RE group and by 22±4% in the RVE group with no significant differences between groups *(P* = 0.85), see Figure 4A.

#### Long-term effects

After the final exercise, endostatin concentrations in the RE group were uniformly greater than concentrations after the initial exercise (time * intervention effect: *P*<0.001, see Figure 4B(i). This long-term effect was not seen in the RVE group (time * intervention effect: *P* = 0.991), see Figure 4B(ii).

### VEGF

#### Acute effects

**Figure 5:**
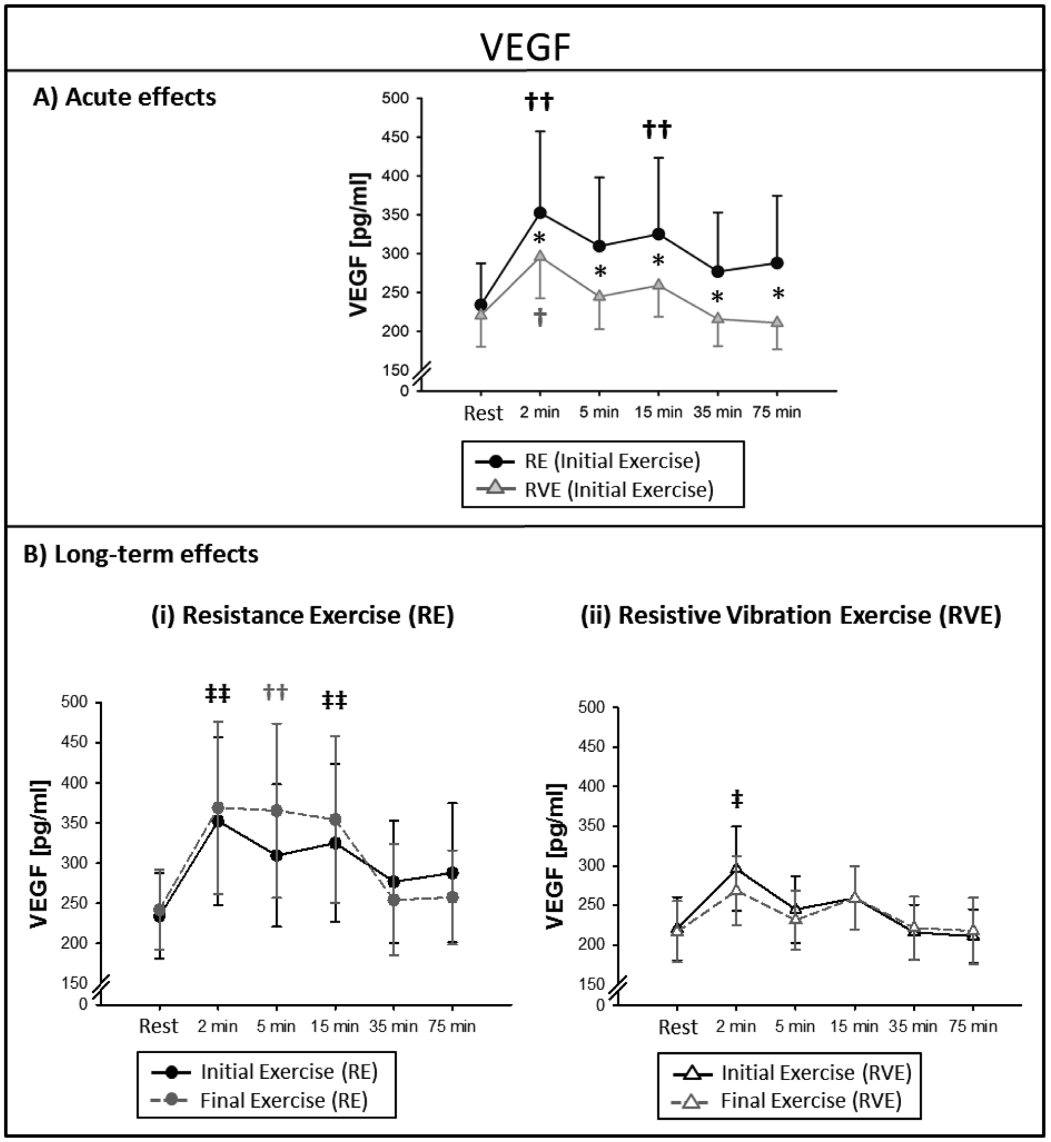
Circulating Vascular Endothelial Growth Factor (VEGF) levels at rest and 2–75 min after exercise. Data points indicate mean serum concentrations (± SEM) at the initial and final exercise sessions of a 6-week exercise intervention (n = 13). Significant differences from resting levels (time effect): ^††^ *P*<0.01; both indicated exercises: ^‡‡^ *P*<0.01. (A) Acute effect of resistance exercise (RE) and resistive vibration exercise (RVE): VEGF was elevated from resting levels 2–15 min after RE and only 2 min after RVE with significantly higher VEGF levels in the RE group. (B) Long-term effects: there were no differences between initial and final exercises in neither group.

In the RE group, VEGF was elevated from resting levels 2–15 min after the initial exercise (time effect: *P*<0.001). In the RVE group, the response differed as this group showed elevated VEGF concentrations only at the time point +*2 min* (time effect: *P*<0.001). VEGF concentrations were significantly higher in the RE group with a 41±16% increase from resting levels compared to the RVE group, which showed a 33±7% increase at the time point +*2 min* (*P* = 0.014). Significantly higher VEGF concentrations in the RE group compared to the RVE were also detected at the remaining time points 5–75 min after exercise termination (*P*-values between 0.02 and 0.004), see Figure 5A.

#### Long-term effects

There were nonsignificant changes in the responses to the exercises after 6 weeks of training, (initial *vs.* final exercise: RE: *P* = 0.520; RVE: *P* = 0.814, see Figure 5B) and VEGF concentrations after the final exercise were also higher in the RE group compared to the RVE group (RE vs. RVE: *P*- values between 0.01 and 0.005).

### Endothelial Cell Proliferation

We used the human serum derived at rest and +*2 min* and +*75 min* after exercise to test the proliferative effect upon human umbilical vein endothelial cells (HUVEC) *in vitro*. These time points were suitable as the angiogenic factors measured via ELISA depicted maximum serum concentrations +*2 min* after exercise termination and concentrations were back at resting levels at the time point+*75 min*. Absorption data detecting BrdU incorporation were normalized to fold increases from resting levels.

**Figure 6:**
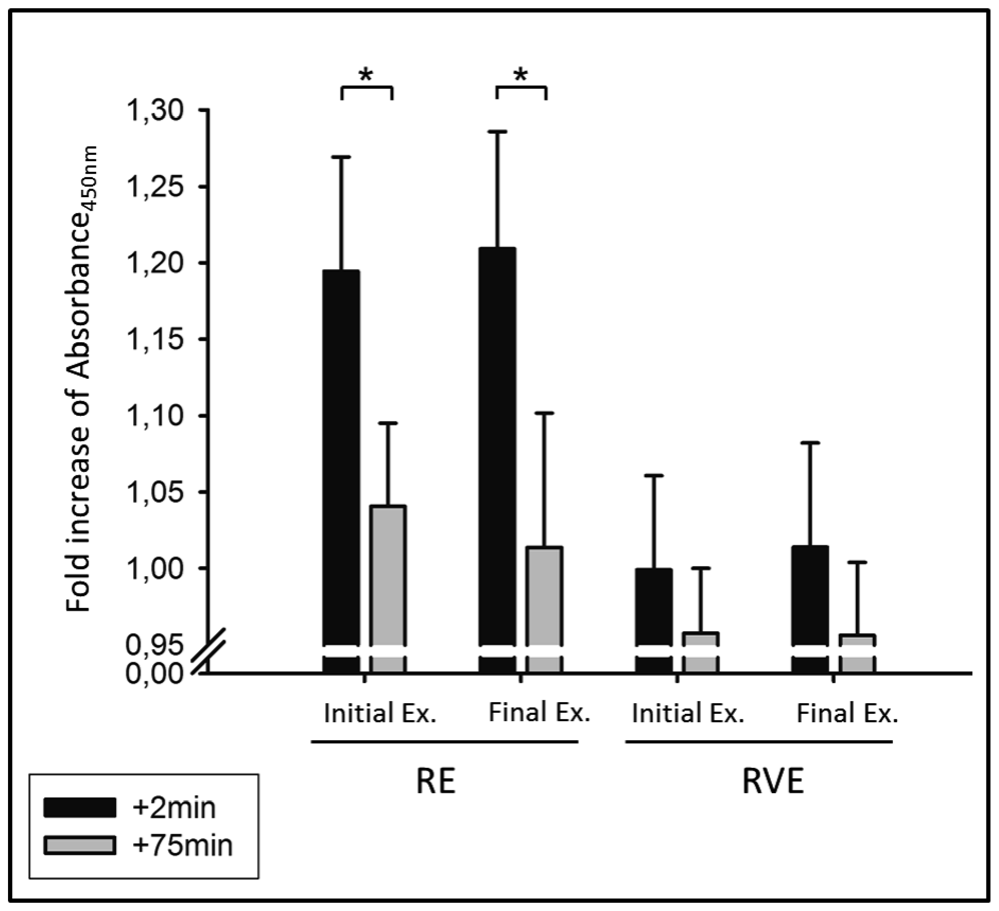
Endothelial cell proliferation measured via BrdU incorporation in human umbilical vein endothelial cells. Bars indicate fold increases of absorbance_450 nm_ of cells incubated with serum derived at rest compared with two minutes (+*2 min*) and 75 minutes (+*75 min*) after exercise. Initial Ex. /Final Ex.: initial and final exercises of a 6-week exercise intervention. RE: resistance exercise, RVE resistive vibration exercise. * time effect: *P* = 0.0171.

Endothelial cells incubated with serum derived at +*2 min* after resistance exercise showed an increased proliferation compared to cells incubated with +*75 min* serum (time effect: *P* = 0.0171). This effect was not seen in the RVE group (time effect: *P* = 0.295). EC proliferation did not differ between cells treated with serum derived after the initial or final exercises in neither group (RE: *P* = 0.94; RVE: *P* = 0.91) and no significant differences between the groups were found (*P* = 0.122), see Figure 6.

## Discussion

To test our hypothesis that superimposing whole-body vibrations to resistance exercise would add a pro-angiogenic stimulus to the training, we evaluated serum concentrations of angiogenic markers *in vivo* and their proliferative capacity upon endothelial cells *in vitro*. Our data indicate that resistance exercise leads to a transient increase of circulating angiogenic markers. Post-exercise serum concentrations of VEGF were higher in the resistance exercise (RE) group compared to the resistive vibration exercise (RVE) group. Additionally, the RE group provoked increased endothelial cell proliferation *in vitro* and showed higher exercise-induced endostatin concentrations. Both effects were elusive in the RVE group.

### MMPs

Degradation of the vascular basement membrane is an initial step in angiogenic sprout formation and allows endothelial cells to migrate into the interstitial matrix in order to form a premature capillary lumen. Matrix metalloproteinases are crucial for extracellular matrix degradation and are thought to be essential for physiological angiogenesis [25]. MMPs have furthermore been implicated in the release and bioavailability of growth factors [26], [27] and play a role in initiating endothelial cell migration and proliferation [28]. Our data show elevated serum MMP-2 levels two minutes after resistance exercise. In the group where whole-body vibrations were superimposed to the exercise, MMP-2 was not elevated after an initial exercise bout but showed an adaptation to long-term training; after 6 weeks of RVE, MMP-2 was elevated above resting levels and concentrations were higher (at rest and post-exercise) compared to the group that had trained without vibrations. This might be a compensatory adaptation to the initial lack of MMP-2. Beyond initiating capillary growth, MMP-2 has furthermore been shown to increase the bioavailability of insulin-like growth factor (IGF) through proteolysis of the IGF binding protein [26], [27]. If this is the case, the observed increases in circulating levels of MMP-2 after six weeks of RVE might reflect an increased IGF-associated anabolic stimulation.

The presented MMP-9 data showed a prolonged increase compared to MMP-2 and MMP-9 was elevated from resting levels until 15 min after both exercise regimes with no detectable long-term effect. A previous study measuring serum MMP-9 concentration pre and post eight weeks of resistance training *vs.* eight weeks of callisthenic training report an increase in the acute MMP-9 response after eight weeks of training only in the callisthenic group [29]. A study on downhill running showed no acute changes in serum MMP-2 but increased serum MMP-9 levels immediately after exercise [30]. Running step tests increased plasma MMP-2 one hour post-exercise whereas plasma MMP-9 was increased immediately after exercise with decreased resting MMP-9 levels after six months of training [7].

Together with the present findings, these data suggest that MMP-responses to acute and long-term training interventions highly depend on workload, volume and contraction form of the exercise. The exposure to different mechanical stimuli seems to foster extracellular matrix remodeling in divergent ways, revealing a potential role of MMPs in initiating training-specific muscle adaptations. A limitation of the procedure is that the available antibodies do not differentiate between the active and pro-enzyme forms of MMPs and we did not measure serum concentrations of tissue inhibitors of metalloproteinases. Therefore, increased MMP-2 and MMP-9 immunoreactivity does not necessarily indicate an increased enzymatic activity.

### Endostatin

Our data show that circulating endostatin was elevated from resting levels 2–15 min after a bout of resistance exercise with no additional effect of superimposed vibrations. Previous studies report prolonged elevations of circulating endostatin compared to the time curves we observed: elevations in plasma from 1 h [31] until 6 h post exercise [12] have been reported after endurance exercise. After 90 min of cycling exercise, Suhr and colleagues [13] found endostatin to be elevated in plasma 0–60 min after exercise termination and superimposing vibrations to this exercise type shortened the elevation from baseline levels to 0 min after exercise, which is an effect of superimposed vibration we did not observe in the present study.

Although we did not see any long-term adaptations in basal endostatin levels, as previously reported for endurance training [7], the response was altered after the 6-week training intervention. Endostatin concentrations in serum were acutely higher after 6 weeks of training and this adaptation was specific for the RE group. Thus, superimposed vibrations seemed to inhibit this biological adaptation to long-term training. Due to endostatin's complex signaling functions, it is not a simple task to interpret the physiological impact of elevated endostatin concentrations after exercise. Initially, endostatin was described as an anti-angiogenic protein [10] capable of inducing apoptotic signals in endothelial cells [32] and to inhibit EC migration, -proliferation and tube formation [33]. Conversely, it was later shown that endostatin has both pro- and anti-angiogenic functions depending on its concentration and the proliferation status of endothelial cells [11]: towards the running opinion, Schmidt and colleagues [11] showed that endostatin concentrations of 50 ng/mL induced EC proliferation and migration with no induction of apoptosis; whereas concentrations of 1000 ng/mL and above had the contrary effect. Based on these data, the endostatin concentrations we reported in the present study (90-140 ng/mL) lie close to the concentrations that were considered as a pro-angiogenic range. Thus, the observed increase in endostatin response after 6 weeks of training (RE only) might reflect a pro-angiogenic long-term training adaptation, which is inhibited by superimposed vibrations.

The acutely elevated endostatin levels seem to have a critical function during exercise. As recently demonstrated by our group, endostatin induces the release of the vasodilator NO in endothelial cells [34]. The acute exercise-dependent endostatin release therefore seems to be essential to activate signaling pathways that result in peripheral vasodilation and consequently improves oxygen delivery to working skeletal muscles to maintain the muscle performance capacity.

### VEGF

The process of endothelial cell proliferation is mediated mainly by Vascular Endothelial Growth Factor (VEGF), a potent endothelial cell mitogen [14]. Exercise leads to increases of VEGF protein in muscle tissue [31] and VEGF has shown to be essential for exercise-induced angiogenesis in skeletal muscle [18]. VEGF serum concentrations were shown to be decreased [12], [31] or elevated [35] after endurance-type exercise. Our data are to our knowledge the first that reveal acute increases of circulating VEGF immediately after resistance-type exercise. We could show that VEGF was elevated in serum 2–15 minutes after resistance exercise, whereas superposition of vibrations to the exercise shortened this response to only two minutes after exercise and provoked significantly lower VEGF concentrations compared to the group that trained without vibrations. As we did not measure VEGF expression in muscle tissue, this finding gives rise to multiple possible explanations. First, decreased circulating VEGF could indicate that more VEGF is still held and active in the tissue and has not been washed out into the blood. Second, reduced circulating VEGF upon vibration exposure could indicate that whole-body vibrations in some way prevented VEGF secretion or release in muscle tissue, which would indicate that superimposing vibrations would not be beneficial for a potential activation of angiogenic signaling in skeletal muscle. Third, VEGF is produced in many cell types and the increased circulating VEGF might also derive from a systemic exercise effect which is not related to muscle tissue and could indicate enhanced endothelial regeneration, which would reflect a beneficial effect of resistance exercise that was inhibited by superimposed vibrations.

In a previous study in our lab, the effect of high-intensity cycling exercise with and without whole-body vibrations was evaluated and this study revealed contrary results considering vibration exposure: plasma VEGF levels were only increased in the group where vibrations were superimposed to the exercise stimulus [13]. As previous studies reveal that WBV increase the shear stress in blood vessels [19], Suhr and colleagues concluded that vibration-induced increases in shear stress-stimulated VEGF release as described by Milkiewicz and colleagues [15]. This explanation does not seem to be applicable in the present study, as our data reveal the contrary, i.e. reduced VEGF upon vibration exposure. Thus, whole-body vibration stimulation seems to have differential effects according to the mode it is applied. In the case of endurance cycling exercise, superimposed vibrations might be beneficial for promoting angiogenesis (reflected by increases in VEGF), whereas our data reveal that the contrary seems to be the case for resistance exercise. As exercise times in the aforementioned study (90 min) were much longer compared to the present study (9 min), it might well be that the initial effects of the exercises are comparable but the measured VEGF kinetics may differ due to the time shift in the measurements.

It is well known that levels of angiogenic markers differ according to the type of blood product in which they were measured (serum *vs.* plasma). Previous studies were inconsistent in the type of blood product used and this might contribute to discrepancies between studies.

### Endothelial cell proliferation

One limitation of measuring angiogenic markers in serum is that their site of action resides within the muscle tissue itself and we determine merely the ‘wash-out’ in serum. Consequently, we sought to investigate whether and in which manner elevated serum concentrations would possibly influence endothelial cells *in vitro*, because this model is well-established to test general defined reactions of endothelial cells *in vitro* that might reflect *in vivo* situations.

As all factors showed maximum concentrations +*2 min* after exercise and were back at resting levels +*75 min* after exercise, we chose to treat human umbilical vein endothelial cells (HUVEC) with serum derived from these time points. We found that endothelial cells incubated with serum derived +*2 min* after RE showed increased proliferation compared to cells incubated with serum derived+*75 min* after exercise. This effect was not seen in the RVE group. VEGF was the only angiogenic factor that showed group-specific differences after exercise (see Figure 5A). VEGF serum concentrations were higher +*2 min* after RE ([352±104 pg/mL] after initial- and [369±107 pg/mL] after final exercise) compared to +*2 min* after RVE ([280±50 pg/mL] after initial- and [268±43 pg/mL] after final exercise), which may be an explanation for the group-specific differences in cell proliferation. The recommended VEGF concentration for HUVEC culture is 500 pg/mL (Endothelial Cell Growth Medium KIT, #C-22110, PromoCell, Heidelberg, Germany), which lie close to the VEGF concentrations we measured in the RE group. However, there are various additional factors that were not measured in the present study that, however, could have influenced HUVEC proliferation, i.e. basic Fibroblast Growth Factor [36], epidermal growth factor (EGF) or heparin-binding EGF-like growth factor [37].

Thus, our data, with certain limitations, reveal that superimposed whole-body vibrations to resistance exercise leads to decreased endothelial cell proliferation, probably due to decreased release or expression of VEGF. Considering long-term adaptations, we did not find any differences in HUVEC proliferation when comparing initial and final exercise sessions. Despite acutely higher endostatin levels during the final exercise in the RE group and higher MMP-2 concentrations in the RVE group, these effects were not reflected by increased cell proliferation during the final exercise.

### Comparison of Time curves

When comparing the time curves of MMP-9 with VEGF and endostatin, it seems that the exercise-induced increase of MMP-9 is paralleled by VEGF and endostatin. First, all factors were increased 2-15 min after exercise and second, all three factors show increased mean concentrations after 6 weeks of training (although only significant for endostatin), see Figure 3B(i), 4B(i) and 5B(i). Conversely, the factor MMP-2 showed different kinetics as it was elevated only for two minutes after exercise and the long-term adaptation that was seen for MMP-2 in the RVE group was specific for MMP-2 and did not affect any of the other factors. In sum, these observations indicate that MMP-9, VEGF and endostatin seem to be interdependent, whereas MMP-2 seems to be differentially regulated. Our data are in line with previous observations in cell culture which showed that MMP's are capable of inducing VEGF release [38]. Moreover, the presented data confirm a previous finding in which the authors described that MMP-9 was more prone to release VEGF compared to MMP-2 *in vitro* and that that MMP-2 regulation occurred independently of VEGF signaling [28]. The parallel increase of MMP-9 and endostatin confirms that endostatin is proteolytically released by MMP's, as described previously [8] and our data hint to MMP-9 playing a larger part in this release compared to MMP-2, at least after bouts of resistance exercise.

In summary, our data show that RE leads to transient increases in circulating pro-angiogenic markers and furthermore, endothelial cell proliferation *in vitro* is increased by factors in serum obtained acutely after RE. Superimposing vibrations to resistance exercise decreases post-exercise circulating VEGF concentrations, which supposedly results in reduced endothelial cell proliferation *in vitro*. Six weeks of RE increased endostatin concentrations acutely after exercise, which is considered as a pro-angiogenic adaptation which was prevented by training with superimposed vibrations. In other words, the presented data suggest that superimposing a vibrations stimulus to resistance exercise might not be beneficial for triggering angiogenic-inducing signaling pathways in skeletal muscle.
